# Evaluation of bladder stimulation as a non-invasive technique for urine collection to diagnose urinary tract infection in infants under 6 months: a randomized multicenter study (“EE-Sti.Ve.N”)

**DOI:** 10.1186/s13063-019-3914-2

**Published:** 2019-12-27

**Authors:** D. Demonchy, C. Ciais, E. Fontas, E. Berard, J. Bréaud, P. S. Rohrlich, F. Dubos, C. Fortier, J. Desmontils, A. L. Hérisse, D. Donzeau, H. Haas, A. Tran

**Affiliations:** 1 Pediatric Emergency Department, Hôpitaux pédiatriques de Nice CHU-Lenval, Nice, France; 2 Délégation à la Recherche Clinique et à l’Innovation, Université Côté d’Azur, Centre Hospitalier Universitaire de Nice, Nice, France; 3 Pediatric Nephrology Unit, Université Côte d’Azur, Centre Hospitalier Universitaire de Nice, Nice, France; 4 Department of Pediatric Surgery, Hôpitaux pédiatriques de Nice CHU-Lenval, Nice, France; 5 Department of Hematology, Université Côte d’Azur, Centre Hospitalier Universitaire de Nice, Nice, France; 6 Pediatric Emergency Unit and Infectious Diseases, Université de Lille, Centre Hospitalier Universitaire de Lille, Lille, France; 7 Department of Pediatrics, Centre Hospitalier Général d’Antibes, Antibes, France; 8 Department of Pediatrics, Centre Hospitalier Général de Grasse, Grasse, France

**Keywords:** Bladder stimulation, Urinary tract infection, Contamination, Urine specimen collection, Infant, Feasibility study

## Abstract

**Background:**

Febrile urinary tract infection (UTI) is common in infants and needs to be diagnosed quickly. However, the symptoms are non-specific, and diagnosis can only be confirmed after high quality urinalysis. The American Academy of Pediatrics recommends suprapubic aspiration (1–9% contamination) and urinary catheterization (8–14% contamination) for urine collection but both these procedures are invasive. Recent studies have shown a new non-invasive method of collecting urine, bladder stimulation, to be quick and safe. However, few data about bacterial contamination rates have been published for this technique. We hypothesize that the contamination rate of urine collection by bladder stimulation to diagnose febrile UTI in infants under 6 months is equivalent to that of urinary catheterization.

**Methods/design:**

This trial aims to assess equivalence in terms of bacterial contamination of urinary samples collected by urinary catheterization and bladder stimulation to diagnose UTI. Seven hundred seventy infants under 6 months presenting with unexplained fever in one of four Pediatric Emergency Departments in France will be enrolled. Each child will be randomized into a bladder stimulation or urinary catheterization group. The primary endpoints will be the validity of the urine sample assessed by the presence of contamination on bacterial culture.

**Conclusion:**

A high recruitment rate is achievable due to the high prevalence of suspected UTIs in infants. The medical risk is the same as that for routine clinical care as we analyze patients with isolated fever.

If our hypothesis holds true and the rate of urine contamination collected by bladder stimulation is acceptable, the infants included in the study will have benefited from a non-invasive and reliable means of collecting urine.

**Trial registration:**

ClinicalTrials.gov, NCT03801213. Registered on 11 January 2019.

## Background

Urinary tract infection (UTI) is common in infants and needs to be diagnosed quickly. The risk for UTI before the age of 2 years is about 1–4% in boys and 3–8% in girls [1, 2]. A delay in diagnosis can lead to severe complications: renal scarring (7.2–15%), high blood pressure (0.7–35%), kidney failure (0.4%) and severe sepsis (5.6–9.3%) [1, 3, 4]. In infants, as symptoms are non-specific (unexplained fever of 38 °C or higher, vomiting, lethargy, irritability, jaundice, poor feeding, abdominal pain, hematuria), the diagnosis of UTI requires a good-quality urine sample [5, 6] that is not easy to obtain before potty training.

In routine practice, different techniques are used to collect urine samples: suprapubic aspiration, urinary catheterization, urine collection bag and clean-catch urine. The American Academy of Pediatrics (AAP) recommends suprapubic aspiration (1–9% bacterial contamination) [7–9] and urinary catheterization (8–14% bacterial contamination) [7–9] but these techniques are invasive and painful. The sterile bag is a non-invasive method of urine collection, but has high rates of bacterial contamination (26–62%) [4, 6] leading to unnecessary antibiotic treatment. Finally, clean-catch urine provides an acceptable urine sample to diagnose UTI according to the recommendations (13–27% of bacterial contamination) [8, 10–13] but this method is only possible for potty-trained children.

Recent studies have shown that bladder stimulation, which consists of pubic tapping and lumbar massage, could be a new, effective, non-invasive and safe method of collecting urine in infants. Tran et al. [14] showed bladder stimulation to be an efficient technique to obtain midstream urine in 142 infants under walking age. The success rate decreased with age from 88.9% (newborns) to 28.6% (> 1 year) (*p* = 0.0001) and with weight, from 85.7% (< 4 kg) to 28.6% (> 10 kg) (*p* = 0.0004). Altuntas et al. [10] included 127 term newborns in a study collecting urine by bladder stimulation and lumbar paravertebral massage. The success rate of urine collection was 78% within 5 min of starting the stimulation maneuvers. Herreros et al. [15] showed good sensitivity and specificity of urine cultures obtained using bladder stimulation in 60 infants < 90 days old and a low contamination rate (5%).

Microscopy and culture are the preferred methods for diagnosing UTI [6]. However, we do not have data from robust studies on the bacterial contamination rate in urine samples using bladder stimulation in infants < 6 months of age. In this randomized, multicenter, prospective clinical trial, we hypothesize that urine contamination rates from bladder stimulation or urinary catheterization are equivalent to the diagnosis of febrile urinary tract infection in infants < 6 months of age. If our hypothesis holds true and the rate of urine contamination collected by bladder stimulation is acceptable, the infants included in the study will have benefited from a non-invasive and valid means of collecting urine.

## Methods/design

### Aim of the trial

The aim of the trial is to assess equivalence, in terms of bacterial contamination, of two techniques for urine sample collection - bladder stimulation versus urinary catheterization - in infants under 6 months of age, suspected to have UTI.

### Primary and secondary outcomes

The primary outcome is bacterial contamination, defined as the growth of two or more micro-organisms, or the presence of a non-uropathogenic strain of bacteria (lactobacilli, Staphylococcus coagulase-negative, Corynebacterium), or bacteriuria with fewer than 10^4^ colony-forming units (CFU)/mL in urine collected by urinary catheterization and fewer than 10^5^ CFU/mL in clean-catch urine collected by bladder stimulation, or leukocyturia with fewer than 10^4^/mL [6, 7, 16–18]. These criteria were chosen based on the assumption that the clean-catch urine collected by bladder stimulation was at least as good as the clean-catch urine obtained during urination of continent infants. The principle of the clean-catch urine being to avoid contamination by commensal flora of the urethra, there should be no difference in quality between these two modes of collection. A sterile culture result (absence of germ) defines the absence of UTI and the absence of contamination.

The bacterial contamination rates for the two techniques will be calculated as the ratio between the number of samples presenting contamination after cytobacteriological examination of the urine (CBEU) and the number of urine samples undergoing CBEU in each group.

Secondary outcomes will assess the discomfort of the technique, the diagnostic performance of the urinary dipstick using both techniques of urine sample collection, and the risk factors associated with bladder stimulation failure.

The discomfort of the technique will be evaluated at different times of the procedure using the EVENDOL [19] (a validated tool to evaluate pain in infants from birth to 7 years old in an emergency room setting): time 1: before disinfection; time 2: for bladder stimulation, the discomfort will be evaluated at the start of the technique and for urinary catheterization when the catheter is introduced into the urethra; time 3: for both techniques, the discomfort will be evaluated 1 min after the end of the maneuvers (after miction for bladder stimulation and removal of the catheter for urinary catheterization, and once the infant has been put back in the parent’s arms). For children who will have undergone two stimulation maneuvers (see “Intervention” section below), the scores registered during the second maneuver will be considered for analysis.

The diagnostic performance of the urinary dipstick will be established through sensitivity (Se), specificity (Sp), positive predictive values (VPP), and negative predictive values (VPN), and accuracy taking the urine culture as the gold standard. A “positive urinary dipstick” is defined by the presence of leukocytes (at least “low” or “+”) and/or positive nitrites.

Risk factors potentially associated with failure of the bladder stimulation technique (no urine or quantity < 2 mL) that we will study are: pain, weight (in kilograms), sex, age (in months), time since last meal (time between urine sample collection and the last meal, in minutes), and time since last urination (time between urine sample collection and the last urination, in minutes).

### Design/setting/participants

This is a prospective, multicenter, randomized clinical trial conducted in four Pediatric Emergency Departments at the Children’s University Hospital of Nice (France), the University Hospital of Lille (France), the General Hospital of Grasse (France), and the General Hospital of Antibes (France). The regional ethics committee (*Comité de Protection des Personnes Sud-Méditerranée* V) has approved the trial protocol “EE-Sti.Ve.N” (ID RCB: 2019.01.09 ter_18.12.18.52954). The study has been registered in the ClinicalTrials.gov protocol registration system (NCT03801213). Infants (*N* = 770) under the age of 6 months with suspected UTI will be included, 480 in the bladder stimulation group and 290 in the urinary catheterization group.

Inclusion criteria are infants under the age of 6 months requiring urine sample collection for the diagnosis of an UTI and signed parental consent. Exclusion criteria are: vital distress signs and contraindication to bladder catheterization (external genitalia or bladder malformation) (Table 1).

**Table 1:** List of all inclusion and exclusion criteria

Inclusion criteria	Exclusion criteria
✓ Infants 6 months of age ✓ Suspected febrile urinary tract infection: ○ fever with temperature > 39 °C without symptoms ○ or fever with temperature > 38 °C and uropathy or urinary tract infection ○ or fever with temperature > 38 °C and < 3 months of age ○ or fever with temperature > 38 °C and duration > 48 h ○ or fever with temperature > 38 °C with signs of sepsis ✓ Signed parental consent	✗ Presence of vital distress signs ✗ Presence of a contraindication to bladder catheterization (external genitalia or bladder malformation)

**Fig. 1:**
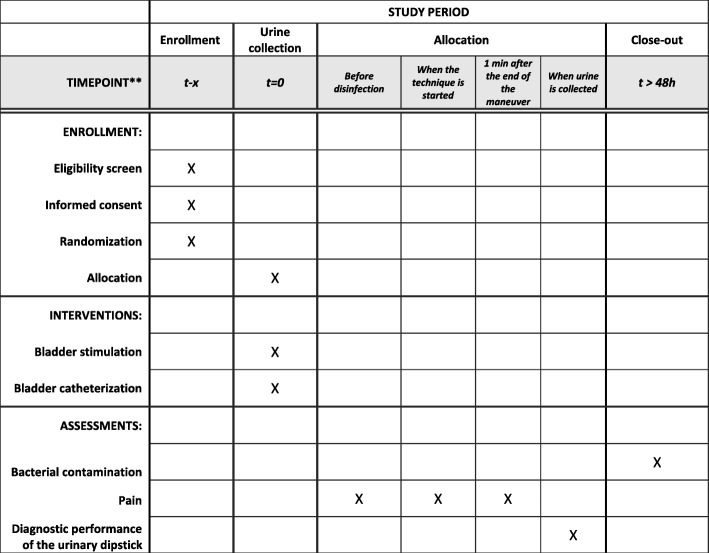
Schedule of enrollment, interventions, and assessments: Standard protocol items: recommendations for interventional trials (SPIRIT)

The total duration of recruitment will be 2 years. The duration of patient participation will be 1 day (Fig. 1).

### Intervention

**Fig. 2:**
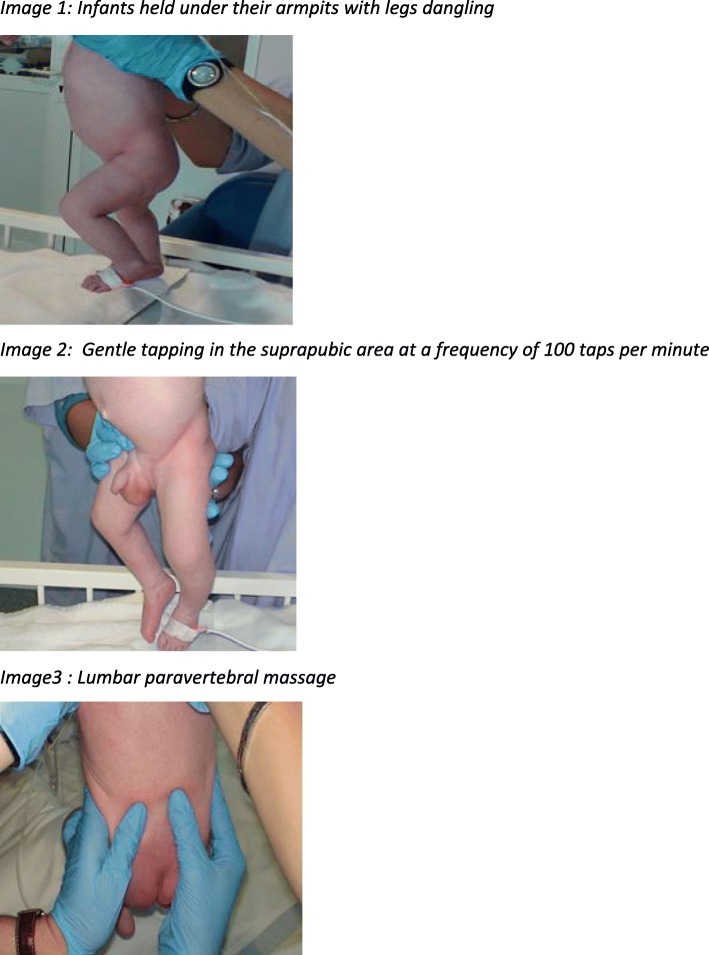
Bladder stimulation and paravertebral massage

The bladder stimulation technique is performed after genital cleaning with a 2% castile soap, which is part of the sterile clean-catch urine collection cup kit. For the technique, infants will be held by a parent under their armpits over the bed, boys with legs dangling and girls with hips flexed (Fig. 2). The nurse or technician will then perform the bladder stimulation maneuvers of gentle tapping in the suprapubic area at a frequency of 100 taps per minute alternated with lumbar paravertebral massage. These two maneuvers will be alternated every 30 s until the urine is obtained, with a maximum duration of 3 min. In the case of failure, the maneuver can be repeated after 20 min. In the case of failure of the two attempts, urine will be collected by urinary catheterization.

Urinary catheterization will be carried out by a nurse or technician according to the usual recommendations and routine practice. These recommendations and practices will be verified and validated with each of the centers at the initial training meeting.

After collection, the urine will immediately be transferred to each center’s laboratory in urine culture tubes. All the samples will be initially analyzed by Multistix® 10 SG test strips (Siemens) to determine nitrite and leukocyte esterase. The urine will be inoculated into the chromogenic medium *UriSelect*®*4* (Bio Rad) with 10 μL calibrated loops. Samples are then incubated at an ambient air temperature of 37 °C. Urine culture results are evaluated after 24 h. Samples without any growth or fewer than the minimum CFUs will be considered to be negative. The bacteria growing in positive cultures will be assessed by biochemical reaction. If evaluation after 24 h of incubation is inconclusive, the plates will be checked again after 48 h.

### Randomization

**Fig. 3:**
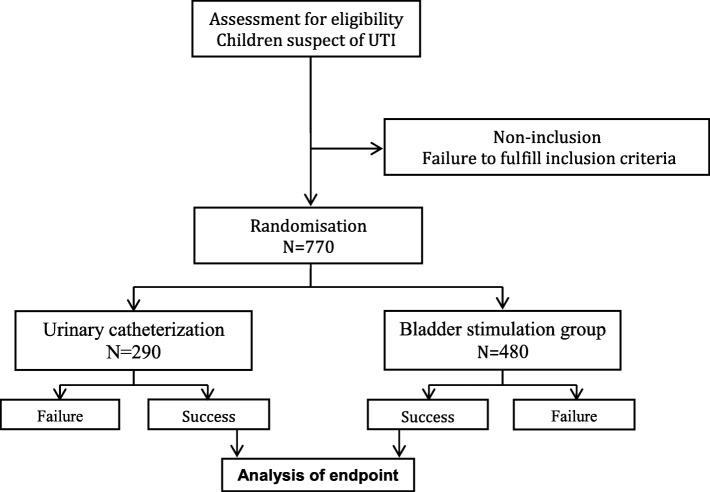
Flow chart. UTI, urinary tract infection

The infants will be randomized into one of the two study groups, the intervention group (urine collection by bladder stimulation) and the control group (urine collection by bladder catheterization) (Fig. 3).

The investigator will proceed to randomization once written informed consent is obtained from both parents, and after the selection criteria have been checked. Randomization will be integrated in the electronic case report form (e-CRF) devised specifically for the study with Open Clinica® software. Using their personal access details to log in, the investigator will provide the necessary patient information (i.e., the first letter of their first and last names and their date of birth) for random allocation to treatment by the online randomization module (Inclusio®). Patients can be randomized around the clock, 7 days a week. The treatment group and inclusion number for the patient will then be relayed to the investigator. The patient’s trial records will be created automatically, allowing data to be entered.

Randomization will be performed centrally at the Department of Clinical Research and Innovation (DRCI) of Nice University Hospital. The randomization will be stratified by each participating center. The randomization lists will be compiled using nQuery Advisor® v 7.0 software, which allows unbalanced group size (block technique; the size of the blocks is not specified to preserve randomization).

### Power calculation

To date, no data are available on contamination rates in urine samples collected by bladder stimulation. In the literature, the average contamination rates associated with bladder catheterization and clean-catch urine are reported as about 10% and 20%, respectively [8, 10, 15].

As previously discussed, we assume that the contamination rate with bladder stimulation is of the same order of magnitude as with the clean-catch urine. We thus hypothesize a contamination rate of 20% with bladder stimulation. Our study hypothesis is that the contamination rates with the techniques studied - urinary catheterization and clean-catch urine through bladder stimulation - are equivalent.

Following AAP guidelines [7], which specify that clean-catch urine is an acceptable technique of urine collection in the diagnosis of UTI, we are interested in the use of clean-catch urine collection up to a 30% contamination rate threshold in view of its non-invasiveness. Indeed, we consider an equivalence limit of 20% compared to the contamination rate with urinary catheterization to define equivalence between the two techniques.

The tested statistical hypothesis, will be:

$$ {H}_0:\mid {P}_{Urinary\kern0.34em Catheterization}-{P}_{Bladder\kern0.17em Stimulation}\mid \ge 0.20 $$

Versus

$$ {H}_1:\mid {P}_{Urinary\kern0.34em Catheterization}-{P}_{Bladder\kern0.17em Stimulation}\mid <0.20 $$

Assuming a type I error rate of 5% and power of 90%, the sample size required is 260 children per arm (nQuery Advisor® v 7.0). From our preliminary study we anticipate that the bladder stimulation technique will fail in 40% of the children. Therefore, we need to enroll 434 patients to reach the expected sample size in this group. If we recruit 10% patients more to allow for incomplete data or technical problems, we need to include 480 patients in the bladder stimulation arm and 290 in the bladder catheterization arm for a final sample size of 770 infants.

### Statistical analysis

#### General considerations

Statistical analysis will be performed by the biostatistician of the DRCI at the University Hospital of Nice. Before each analysis, the conditions under which the tests were applied will be verified. The various tests will be considered significant at a threshold of 5% (unless otherwise specified). Continuous variables will be described using the number of observations (*N*), arithmetic mean (mean), standard deviation, minimum (min), median (med), and maximum (max) values. Categorical variables will be summarized by absolute (*N*) and relative frequencies (percentage (%)). The statistical analysis will be performed using SAS Enterprise Guide 7.1 software (Copyright (c) 2017 by SAS Institute Inc., Cary, NC, USA).

#### Patient disposition

In accordance with the Consolidated standards of reporting trials (CONSORT) 2010 statements, we will present a patient flow diagram for the various phases of the study: enrollment, allocation, follow up, and analysis.

#### Demographic and baseline characteristics

Baseline demographics and clinical characteristics data will be listed and summarized by group. As recommended by the CONSORT guidelines, the principal characteristics of the patients will be compared between the two groups at inclusion, but no statistical analysis of this will be performed. The comparability of the two arms will be assessed clinically rather than statistically.

#### Missing data

No data replacement has been planned as we expect few missing data due to the short duration of patient participation.

#### Model adjustment

The statistical models that are constructed will be first adjusted by center, which is a stratification parameter of randomization. To take into account any potential confounders that may persist despite the randomization, the adjustment may be completed by factors that would be clinically imbalanced between the groups at baseline.

#### Analysis of the primary endpoint

The contamination rate will be presented in each group with its 95% confidence interval (CI). Equivalence will be assessed if the exact 95% CI of the difference between the contamination rates is entirely included in the interval (− 20%; 20%).

The rate of failure (no CBEU performed) of each technique will also be presented both globally and according to the reason for failure (urine not collected, insufficient volume of urine, technical problem in the laboratory, etc.). In the bladder stimulation group, the failure rate will be given after two attempts and the proportion of parents refusing a second attempt will be recorded.

As recommended for equivalence studies, the main analysis will be run per-protocol. Indeed, as a high rate of failure is expected in the bladder stimulation group (around 40%), replacement of missing CBEU data could lead to biased comparison of the techniques. We believe it is more appropriate to discuss our results and the relevance of bladder stimulation in light of the observed failure rates.

#### Analysis of secondary endpoints

Discomfort (EVENDOL score) during the maneuvers will be compared between the two groups using analysis of covariance with EVENDOL during the maneuver as the dependent variable and the group as the variable of interest. The model will be adjusted on the EVENDOL score at baseline and the center (stratification parameter). Other potential confounding factors may be considered as described previously (see “Model adjustment”). The same analysis will be rerun to compare discomfort after the maneuver.

Diagnostic performance for detecting UTI by dipstick using urine collected through catheterization or stimulation will be assessed taking urine culture as the gold standard. Se, Sp VPP, and VPN will be calculated with 95% CI, using Wilson’s score method. Accuracy will be also calcualted. Only patients with an evaluable dipstick and CBEU will be included in this analysis.

Associations between potential risk factors (see “Primary and secondary outcomes”) and bladder stimulation success will be studied using univariable and multivariable logistic regression models with the success of the stimulation as the dependent variable. Backward elimination selection will be used in the multivariable analysis [20]. Finally, the multivariate model will be adjusted as planned (see “Model adjustment”). The OR and 95% CI will be calculated. This analysis will be performed on all patients in the experimental group who underwent bladder stimulation: infants who do not undergo a second attempt of urine collection after failure of a first attempt will not be included in this analysis.

### Data management

#### The database

The data for the study will be captured in the e-CRF which will be devised by the Data Manager of the DRCI using OpenClinica® software. Specification of parameters and the implementation of the e-CRF for data collection, including user training, will be the responsibility of the DRCI.

The investigators and clinical research assistants in each center will collect the data and enter them directly into the e-CRF. The data will be securely stored, with specific access rights granted to members of the study team according to their role in the study.

#### Data quality control

Data quality control of the e-CRF will be performed by the Sponsor using the patients’ medical files, during planned monitoring visits by the DRCI’s Clinical Research Officer. Once the final data have been entered, their validity and coherence will be checked by the Data Manager of the DRCI, and any requests for verification issued. Any modifications to the database will be recorded throughout the study, thereby enabling a full audit trail.

At the end of the quality control process, the database will be locked and signed off by the Principal Investigator, the Data Manager, and the Head of the Biometrics Department at the DRCI. No data modification will be possible after this time. The locked database, together with the data management report, will then be transferred to the statistician for analysis.

#### Safety

Any observed side effects related to bladder stimulation or urinary catheterization will be documented throughout the trial period and reported to the Sponsor without delay. These data will be provided for periodical review by the Data and Safety Monitoring Board.

## Discussion

The risk of contracting a UTI before the age of 2 years is high [1, 2] and any delay in diagnosis may expose the infant to severe complications. However, diagnosing UTI in infants is difficult because the symptoms are non-specific: fever with temperature higher than 38 °C for more than 48 h without any source, vomiting, abdominal pain, apathy, irritability, anorexia, and jaundice [5, 21]. The gold standard to diagnose a UTI requires CBEU which implies collecting a urine sample by fairly invasive and painful techniques for infants before they are potty trained. Improperly collected specimens or incorrect interpretation of test results may contribute to under or over diagnosis of UTI. Current AAP recommendations include collection by suprapubic aspiration (1–9% bacterial contamination) [7–9] and urinary catheterization (8–14% contamination) [7–9]. The clinician’s choice of technique should be guided as follows: the best quality of the urine sample, the least invasive, the safest and quickest technique, and potentially the least expensive.

Our team [14] have previously assessed a bladder stimulation technique to obtain a clean-catch urine sample in infants, with a success rate decreasing with age from 88.9% (newborns) to 28.6% (> 1 year). These findings justify why we have decided to include infants under 6 months in the current study.

Few studies have compared bladder stimulation with other techniques. Herreros et al. [15] compared a standardized clean-catch stimulation technique and bladder catheterization in a small sample of 60 infants under 90 days old. Clean-catch technique sensitivity was 97% (95% CI 8–100%) and specificity was 89% (95% CI 65–98%). The contamination rate of clean-catch samples was lower (5%) than the contamination rate of catheter samples (8%).

In the present study, we hypothesize that bladder stimulation is a technique for obtaining urine with a contamination rate equivalent to that obtained by urinary catheterization, in the diagnosis of febrile UTI in infants under 6 months of age. We aim to demonstrate equivalence in a large sample of 770 infants.

However, our study has a limitation. Ideally, clean-catch urine by bladder stimulation should be compared with urine obtained by suprapubic aspiration or urinary catheterization in the same infant [22, 23]. However, not only would this method be time consuming but, more importantly, it would be quite difficult to obtain parental consent for ethical considerations.

Bladder stimulation is a simple, non-invasive, and safe technique for collecting urine in an infant suspected of having a UTI with a significant success rate (60% in infants under 6 months) and a reasonable collection time in a pediatric emergency unit. If our hypothesis holds true, the infants included in the bladder stimulation group of the study will have benefited from a non-invasive and valid means of collecting urine. Moreover, this new method of collecting urine would be better accepted by the parents than the invasive alternatives.

### Trial status

The regional ethics committee (*Comité de Protection des Personnes Sud-Méditerranée* V) approved the trial protocol (ID RCB: 2019.01.09 ter_18.12.18.52954) on 13 May 2019. Recruitment will start in September 2019 and is estimated to be completed in 2021.

## Supplementary information

**Additional file 1.** SPIRIT 2013 Checklist: Recommended items to address in a clinical trial protocol and related documents*.

**Additional file 2.** informed consent form.

## Data Availability

Not applicable.

## Abbreviations

AAP: American Academy of Pediatrics
CBEU: Cytobacteriological examination of urine
CRA: Clinical research associate
DRCI: Delegation for Clinical Research and Innovation of Nice University Hospital
e-CRF: Electronic case report form
UTI: Urinary tract infection
