# Supplementary material for: Evaluation of bladder stimulation as a non-invasive technique for urine collection to diagnose urinary tract infection in infants under 6 months: a randomized multicenter study (“EE-Sti.Ve.N”)
Source: Trials. 2019 Dec 27;20:783. doi: 10.1186/s13063-019-3914-2 (PMC6935056; doi:10.1186/s13063-019-3914-2)
Supplement: Supplementary file 2 — Additional file 2. informed consent form. [file 13063_2019_3914_MOESM2_ESM.docx]

***« Evaluation de la technique de stimulation vésicale comme moyen d’obtenir des urines de qualité dans le diagnostic d’infection urinaire fébrile chez le nourrisson»***

***EE-Sti.Ve.N***

*Promoteur de l’étude : Fondation LENVAL, Hôpitaux Pédiatriques de Nice CHU-LENVAL,*

*57, Avenue de la Californie – 06200 Nice,*

*Tel : 04 92 03 03 92 ; Fax : 04 92 03 03 44,* [*fondation@lenval.com*](mailto:fondation@lenval.com)

Investigateur coordonnateur: D**r. Diane DEMONCHY – Service des Urgences pédiatriques**

Hôpitaux Pédiatriques de Nice CHU-LENVAL - *57, Avenue de la Californie – 06200 Nice*

Tel: 04 92 03 04 42 - Fax: 04 92 03 03 29

**Version 0.1 du 31/01/2019**

**CONSENTEMENT ECLAIRE – TITULAIRES DE L’AUTORITE PARENTALE**

Je déclare avoir compris le but et les modalités de cette étude, qui m’ont été pleinement expliqués par le médecin signataire

Les informations relatives au principe de l’étude, et son intérêt m’ont bien été communiquées dans la Note d’Information. J’ai eu la possibilité de l’étudier attentivement. Des réponses ont été apportées à toutes mes questions. J’ai disposé d’un délai de réflexion avant de prendre ma décision.

J’accepte de faire participer volontairement mon enfant au **protocole de recherche impliquant la personne humaine « EE-Sti.Ve.N »** dont le promoteur est la Fondation Lenval Hôpitaux Pédiatriques de Nice CHU-Lenval et l’investigateur coordonnateur est le Dr.Diane DEMONCHY du Service de Neurochirurgie pédiatrique. Il m’a bien été précisé que je pouvais refuser de faire participer on enfant à cette étude et que si je souhaitais le faire participer, je pouvais revenir sur ma décision à tout moment. Il m’a également été expliqué que j’ai la possibilité de contacter l’un des investigateurs de l’étude (coordonnées à la fin de la notice d’informations) pour poser des questions à tout moment avant et en cours d’étude. J’ai bien compris que le traitement approprié à sa maladie (état de santé) sera mis en place, sans qu’à aucun moment sa participation ou sa non-participation au protocole de recherche **« EE-Sti.Ve.N »** n’ait une quelconque influence sur son suivi ou sur son traitement.

J’ai été informé(e) :

- que, pour cette étude, le promoteur a souscrit une assurance en Responsabilité Civile auprès de la SHAM, sous le numéro de contrat 105 617.
- que, conformément à l’article L.1121-11 du Code de la Santé Publique l’affiliation à un régime de Sécurité Sociale est obligatoire
- que certaines données nominatives le concernant feront l’objet d’un traitement informatisé dans le respect du secret médical.
- que les données de cette étude, incluant celles portant sur son cas, sont couvertes par le secret professionnel.
- de mon droit de m’opposer au traitement automatisé des données nominatives le concernant.
- que toutes les données resteront confidentielles.
- que, conformément à la loi 2012-300 du 05/03/2012, à la fin de l’étude je peux demander à l’investigateur une synthèse des résultats globaux de la recherche. Il m’a été garanti que toute information nouvelle survenant en cours d’essai me sera transmise.
- de mon droit d’accès et de rectification à ces données directement ou indirectement par l’intermédiaire d’un médecin de mon choix désigné à cet effet ou auprès du délégué de la protection des données (contact.dpo@lenval.com). Je n’autorise leur consultation que par les personnes qui collaborent à la recherche, aux personnes chargées par le promoteur de contrôler la qualité de l’étude ainsi que par un représentant des autorités de santé.
- que cette étude a été déclarée à la Commission Nationale Informatique et Liberté selon l’engagement de conformité MR001.
- Les procédures médicales de cette étude sont conformes aux recommandations nationales et l’étude a obtenu l’accord du Comité de Protection des Personnes__________________, avis délivré le XX/XX/XXXX et l’autorisation de l’Agence Nationale de Sécurité du Médicament et des produits de santé (ANSM) le **05/12/2018**

| **🞏 Mère 🞏 Père 🞏 Titulaire de l’autorité parentale**  **De l’enfant : Nom…………………………………………………Prénom :…………………………………………………**  **Nom:……………………………………………………………….…. Prénom :………………………….………………………………………….** |
| --- |
| **Signature Date : …… / …….. / ………** |

| ***LE* *MEDECIN INVESTIGATEUR***  **Nom et prénom du médecin (en majuscules) :…………………………………………………………….…………….**  **Tél :…………………………………………………………………………………………………**  ***En signant ce formulaire, je confirme que j’ai expliqué l’étude avec exactitude au signataire susmentionné*** |
| --- |
| **Signature Date : …… / …….. / ………** |

*Fait en deux exemplaires dont un remis au(x) parent(s).*
